# Zoonotic pathogens associated with *Hyalomma aegyptium* in endangered tortoises: evidence for host-switching behaviour in ticks?

**DOI:** 10.1186/1756-3305-5-301

**Published:** 2012-12-28

**Authors:** Anamaria I Paștiu, Ioana A Matei, Andrei D Mihalca, Gianluca D’Amico, Mirabela O Dumitrache, Zsuzsa Kalmár, Attila D Sándor, Menelaos Lefkaditis, Călin M Gherman, Vasile Cozma

**Affiliations:** 1 Department of Parasitology and Parasitic Diseases, Faculty of Veterinary Medicine, University of Agricultural Sciences and Veterinary Medicine Cluj-Napoca, Calea Mănăștur 3-5, Cluj-Napoca, 400372, Romania; 2 Department of Microbiology and Parasitology, Faculty of Veterinary Medicine, University of Thessaly, Trikalon 224, PO Box 199, Karditsa, 43100, Greece

**Keywords:** *Hyalomma aegyptium*, *Testudo graeca*, *Borrelia burgdorferi* s.l, *Anaplasma phagocytophilum*, *Ehrlichia canis*, *Coxiella burnetii*

## Abstract

**Background:**

*Hyalomma aegyptium* is a hard-tick with a typical three-host life cycle. The main hosts are Palearctic tortoises of genus *Testudo*. However, other hosts can be used by immature ticks for feeding in natural conditions. Given this complex ecology and multiple host use, the circulation of pathogens by *H. aegyptium* between various hosts can be important from epidemiological point of view. The aim of this study was to evaluate the role of *H. aegyptium* as natural carrier of four important zoonotic pathogens.

**Methods:**

From 2008 to 2011, 448 *H. aegyptium* ticks were collected from 45 Spur-thighed tortoises, *Testudo graeca* in Romania. DNA was extracted individually from each tick using a commercial kit. DNA was examined for the presence of specific sequences of *Borrelia burgdorferi* s.l*., Anaplasma phagocytophilum, Ehrlichia canis* and *Coxiella burnetii* by PCR, according to previously described protocols.

**Results:**

PCR analysis of *H. aegyptium* revealed the presence of *A. phagocytophilum* (18.8%)*, E. canis* (14.1%) and *C. burnetii* (10%). 32.4% of the ticks were infected with at least one pathogen and 9.8% had co-infections. The stages most frequently infected were nymphs (50%) followed by males (33.9%) and females (27%). The number of tortoises which harboured infected ticks was 27/45 examined (60%). From all tested *T. graeca*, 40% harboured ticks infected with *A. phagocytophilum*, 46.7% had ticks infected with *E. canis* and 33.3% had ticks with *C. burnetii*. This study reports for the first time the presence of *A. phagocytophilum* and *E. canis* in *H. aegyptium.*

**Conclusions:**

The presence and relatively high prevalence of three important zoonotic pathogens in *H. aegyptium* raises the question of their epidemiologic importance in disease ecology. As tortoises are unlikely to be reservoir hosts for *A. phagocytophilum* and *E. canis* and both these pathogens are common in *H. aegyptium*, this is an important indication for (1) a possible increased host-switching behaviour of these ticks to competent reservoir hosts (i.e. hedgehogs) and (2) transstadial transmission. Furthermore, if we consider also the presence of *C. burnetii*, we conclude that *T. graeca* and its ticks should be evaluated more seriously when assessing the eco-epidemiology of zoonotic diseases.

## Background

*Hyalomma aegyptium* (Linnaeus, 1758) is a hard-tick with a typical three-host life cycle [1]. The main hosts for adults are Palearctic tortoises of the genus *Testudo*[2]. Hence, the distribution of this tick is restricted to the distribution of the principal hosts: Mediterranean bioregion from the Atlantic coastland of Morocco through Northern Africa, Balkan countries, Middle East, and Caucasus and steppic regions in Central Asia, Afghanistan, and Pakistan [3-5]. However, although rarely reported, other hosts (hedgehogs and hares) can be used by adults for feeding in natural conditions [6,7]. Nevertheless, larvae and nymphs of *H. aegyptium* are less host-specific and feed on various vertebrates: tortoises, lizards, birds, small mammals and even humans [1,8-10].

Given this complex ecology and multiple host use, the transmission of pathogens by *H. aegyptium* between reservoir hosts in natural cycles can be important from an epidemiological point of view. Determining the vectorial capacity of a tick to a certain pathogen is questionable if based only on pathogen detection (mainly by PCR), without experimental trials [11]. Several pathogens were detected in *H. aegyptium*: *Theileria annulata*[12], *Borrelia turcica*[13], *Rickettsia* spp. and *Borrelia burgdorferi* s.l. [14]. Experimental trials are usually long and difficult to perform, hence the need for a preliminary assessment of the carrier status in natural populations. Until now, experimental proof of the vectorial capacity of *H. aegyptium* was shown for several pathogens: *Hemolivia mauritanica*[15], *Hepatozoon kisrae*[16], *Rickettsia aeschlimannii*[17] and *Coxiella burnetii*[18].

In Romania, all stages of *H. aegyptium* were found on only two hosts, the Spur-thighed tortoise, *Testudo graeca* and the Northern White-breasted hedgehog, *Erinaceus roumanicus* and its distribution matches the one of the tortoise host [19]. Reptiles can serve as reservoirs for numerous important pathogens [20,21]. Particularly long-living tortoises could have a potential role in long-term maintenance of natural foci of infectious diseases and their ticks can serve as vectors [22,23]. Moreover, in the case of reptile ticks feeding occasionally on mammal hosts (i.e. in their larval and nymphal stage, as the case of *H. aegyptium*) studies regarding the presence of zoonotic agents are of particular interest because of the potential role of these ticks to maintain and cycle the pathogens in nature.

In Romania, there are few studies on the epidemiology and distribution of zoonotic tick-borne pathogens. In the same geographical area (Tulcea and Constanța County), Mircean et al. [24] reported dogs seropositive for *A. phagocytophilum* and *E. canis* and Majláthová et al. [21] found *Ixodes ricinus* ticks infected with *Borrelia burgdorferi* s.l. Moreover, *H. aegyptium* was shown to be a competent vector for *C. burnetii* under laboratory conditions but the natural role of this tick in the ecology of Q fever was never assessed. Hence, the aim of the present study was to evaluate the role of *H. aegyptium* as natural carrier of four important zoonotic pathogens: *Borrelia burgdorferi* sensu lato (s.l.), *Anaplasma phagocytophilum, Ehrlichia canis* and *Coxiella burnetii*.

## Methods

### Sample collection and study area

**Figure 1:**
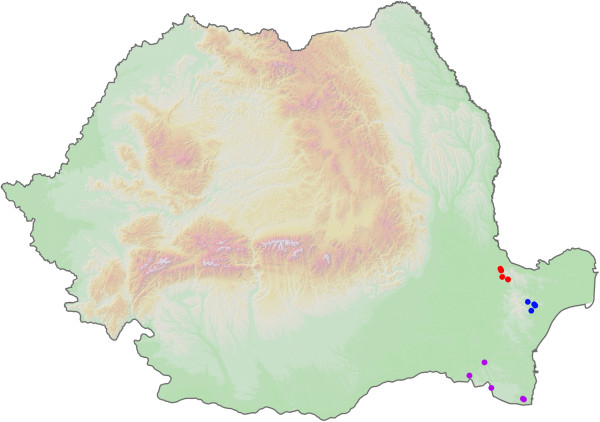
Sample collection sites: Măcin Mountains (red dots), Babadag forest (blue dots) and Constanţa forests (purple dots).

From 2008 to 2011, 448 engorged *H. aegyptium* ticks (2 larvae, 16 nymphs and 430 adults: 304 males and 126 females) were collected from Spur-thighed tortoises, *Testudo graeca ibera* (see Additional file 1 for details on collection sites). No other tick species were found. Tortoises were located and hand caught in their natural environment and released at the spot after tick collection. The animals were captured predominantly in the understory of sub-mediterranean xerophile oak-hornbeam (*Quercus* spp., *Carpinus orientalis*) forests (Figure 1, blue and purple spots) and dry steppe grasslands used as extensive pastures (Figure 1, red spots), with high turnover of small ruminant herding (sheep and goat). A total of 45 tortoises were collected, from 12 localities, all in Dobrogea, SE Romania. Collection was made in the active period of tortoises, from April to June. Individual tortoises were carefully inspected for ticks and all ticks were removed and collected in individual vials. Ticks were stored in ethanol at -20°C until examination. Specific identification of ticks was performed using morphological keys [25] under a binocular microscope.

### DNA extraction and PCR amplification

DNA was extracted individually from each tick using a commercial DNA extraction kit (DNeasy Blood & Tissue Kit, Qiagen), according to the manufacturer’s instructions. Extracted DNA was examined for the presence of specific sequences of *B. burgdorferi* s.l*., A. phagocytophilum* and *C. burnetii* by standard PCR and *E. canis* by nested PCR, with the primers shown in Table 1, according to previously described protocols [26-29]. The amplification was performed in Bio-Rad C1000^TM^ Thermal Cycler. Aliquots of each PCR product were electrophoresed on 1.5% agarose gel stained with SYBR® Safe DNA gel stain (Invitrogen) and examined for the presence of the specific fragment under UV light (Bio-Rad BioDoc-It™ Imagine System). DNA fragment size was compared with a standard molecular weight, 100 bp DNA ladder (Fermentas). Distilled water was used as negative control.

**Table 1:** Targeted genes and list of primers used in this study

**Pathogen**	**Gene**	**Primer sequence (5**^**′**^**-3**^**′**^**)**	**Size of PCR product (bp)**	**Reference**	**Positive controls**
*Borrelia burgdorferi* s.l.	OspA	GGG AAT AGG TCT AAT ATT AGC CCAC TAA TTG TTA AAG TGG AAG T	665 bp	[26]	ATCC BG/V
*Anaplasma phagocytophilum*	msp2	TTG GTC TTG AAG CGC TCG TAATG GAA GGT AGT GTT GGT TAT GGT ATT	420 bp	[27]	*A. phagocytophilum* (dog isolate)
*Ehrlichia canis*	16S rRNA	*Ehrlichia* spp.AGA ACG AAC GCT GGC GGC AAG CCCGT ATT ACC GCG GCT GCT GGC	478 bp	[29]	*E. canis* (dog isolate)
		*Ehrlichia canis* CAA TTA TTT ATA GCC TCT GGC TAT AGCTAT AGG TAC CGT CAT TAT CTT CCC TAT	389 bp		
*Coxiella burnetii*	IS1111	CTC GTA ATC ACC AAT CGC TTC GCAA GAA TGA TCG TAA CGA TGC GC	337 bp	[28]	10^5^*C. burnetii*/ml (culture)

### Statistical analysis

Frequency, prevalence and its 95% confidence interval of *B. burgdorferi* s.l*., A. phagocytophilum, E. canis* and *C. burnetii* DNA and respective co-infections in *H. aegyptium* were established. These parameters were determined according to sex and developmental stage of the ticks (males, females, nymphs and larvae) and geographic location. The difference of prevalence among groups was statistically analysed by chi-squared independence. A *p* value of <0.05 was statistically significant. All statistical analyses were performed using the EpiInfo 2000 software.

## Results

PCR analysis of *H. aegyptium* revealed the presence of three pathogens (*A. phagocytophilum, E. canis* and *C. burnetii*) from the four tested (no samples were positive for *B. burgdorferi* s.l.). From the total number of ticks examined, 145 were infected with at least one pathogen (32.4%; 145/448; CI 95%: 28.1-36.9). The frequency and prevalence of each detected pathogen are shown in Table 2. The highest molecular prevalence was detected for *A. phagocytophilum* (18.8%), followed by *E. canis* (14.1%) and *C. burnetii* (10%). The developmental stages most frequently infected with at least one pathogen were nymphs (50%; 8/16; CI 95%: 24.7-75.3) followed by adult males (33.9%; 103/304; CI 95%: 28.6-39.5) and females (27%; 34/126; CI 95%: 19.5-35.6).

**Table 2:** **Prevalence of *A. phagocytophilum, E. canis and C. burnetii *DNA in *H. aegyptium *collected from *T. graeca***

	***Anaplasma phagocytophilum***		***Ehrlichia canis***		***Coxiella burnetii***	
	**PCR positives/all tested**	**Prevalence % (CI 95%)**	**PCR positives/all tested**	**Prevalence % (CI 95%)**	**PCR positives/all tested**	**Prevalence % (CI 95%)**
**Larvae**	0/2	0 (0.0-84.2)	0/2	0 (0.0-84.2)	0/2	0 (0.0-84.2)
**Nymphs**	8/16	50 (24.7-75.3)	0/16	0 (0.0-20.6)	2/16	12.5 (1.6-38.3)
**Females**	0/126	0 (0.0-2.9)	20/126	15.9 (10.0-23.4)	19/126	15.1 (9.3-22.5)
**Males**	76/304	25 (20.3-30.3)	43/304	14.1 (10.5-18.7)	24/304	7.9 (5.2-11.7)
**TOTAL**	**84/448**	**18.8 (15.3-22.7)**	**63/448**	**14.1 (11.0-17.7)**	**45/448**	**10 (7.5-13.3)**

The prevalence of the co-infections was 9.8% (44/448; CI 95%: 7.3-13.1). The prevalence of co-infections was higher in nymphs (12.5%; 2/16; CI 95%: 1.6-38.3) and males (12.2%; 37/304; CI95%: 8.8-16.5) than in other developmental stages (*p* < 0.00001). The only region where co-infected ticks were found was Măcin Mountains, with 10.9% from the total number of ticks (36/330; CI 95%: 7.9-14.9). The most frequent co-infection type was *A. phagocytophilum*-*E. canis* (43.2%; 19/44; CI 95%: 28.3-59.0), followed by *A. phagocytophilum*-*C. burnetii* (38.6%; 17/44, CI 95%: 24.4-54.5).

The number of tortoises which harboured infected ticks (regardless the pathogen) was 27 out of 45 examined (60%; CI 95%: 44.3-74.3). From all tested *T. graeca*, 18 (40%) harboured ticks infected with *A. phagocytophilum*, 21 (46.7%) had ticks infected with *E. canis* and 15 (33.3%) had ticks with *C. burnetii*. Interestingly, from the 27 tortoises which harboured infected ticks, 18 had ticks infected independently or co-infected with at least two pathogens (Table 3).

**Table 3:** **Number of *Testudo graeca *which harboured ticks infected with pathogens (independent infection of ticks with one or more pathogens, but on the same host)**

**Pathogens**	**A**	**E**	**C**	**A-E**	**A-C**	**C-E**	**A-E-C**	**Negative**
***No. tortoises***	3	6	0	4	3	3	8	18

Legend: A - *Anaplasma phagocytophilum*; E - *Ehrlichia canis*; C - *Coxiella burnetii*.

## Discussion

Ticks are vectors of important pathogens of humans and animals and serve as indicators of infection in nature [30]. The geographical distribution and habitats of several generalist tick species have expanded in the recent years. Major drivers for this trend include land use, climate changes and globalization [31,32]. On the other hand, for certain tick species which are co-distributed with their endangered hosts, like the case of *H. aegyptium*, the trend is a decreasing geographical range [2]. However, in general, a decrease in the availability of natural host populations could lead to host-switching behaviour [33]. As *H. aegyptium* is reported to alternatively feed on various other hosts, mainly during their pre-imaginal stages, evaluation of its zoonotic pathogen burden is of particular interest.

Regarding their role in the ecology of zoonotic infectious diseases, tortoises and their ticks have received significantly less attention compared to mammals and birds. Among small mammals, hedgehogs (*Erinaceus* spp.) are important mainly in synanthropic environments as reservoir hosts for important human pathogens like *A. phagocytophilum*, *Babesia* spp. [34] or *B. burgdorferi* s.l. [35]. As *H. aegyptium* occasionally feeds on hedgehogs and it can potentially attack humans [10], the evaluation of this species as a carrier host for zoonotic pathogens is important.

For *B. burgdorferi* s.l., the main vectors are ticks of genus *Ixodes* and the reservoir hosts, mostly small mammals [36]. Although in this survey there were no *H. aegyptium* positive for the Lyme disease agent, some other studies reported that this tick can feed on reservoir hosts of *Borrelia lusitaniae*[37]. The role of reptiles in the ecology of *B. lusitaniae* was shown in the past by several authors [38,39]. *Borrelia burgdorferi* s.l. is one of the most extensively studied tick-borne pathogens in the world. Hence, there were numerous experimental trials for assessing the vectorial capacity of various ticks. So far, experimental data suggest that only ticks of genus *Ixodes* are competent vectors for the Lyme disease spirochetes [40]. However, Kar et al. [14] found *B. burgdorferi* s.l. in two out of 28 pools of *H. aegyptium* collected from *Testudo graeca* in Turkey. In the present study no ticks were positive for this pathogen, supporting the hypothesis that ticks other than *Ixodes* spp. should not be assumed to serve as bridging vectors for *B. burgdorferi* s.l. or to play any role in the maintenance of these spirochetes in natural cycles [40].

*Anaplasma phagocytophilum*, the agent of human granulocytic anaplasmosis, is vectored in Europe by *Ixodes ricinus* and can infect a wide range of domestic and wild vertebrate hosts, including rodents, horses, dogs and humans [41]. This is the first report of *A. phagocytophilum* in *H. aegyptium* ticks. The relatively high prevalence of *A. phagocytophilum* in this study (18.8%) in fully engorged *H. aegyptium* collected from tortoises can be theoretically caused by two factors: (1) the infection was acquired by ticks during an earlier developmental stage feeding on competent reservoir hosts or (2) tortoises are competent reservoir hosts. The second hypothesis is less probable, as several studies showed that reptiles are not competent reservoir hosts for this pathogen [42,43]. Moreover, surveillance of other ticks parasitic on reptiles yielded negative results [44]. Prevalence of *Anaplasma* spp. in engorged *Hyalomma lusitanicum* and *H. marginatum* collected on domestic mammals in Sicily was much lower (~1%) [45]. All these data (improbable reservoir role of tortoises and relatively high prevalence), suggest that *H. aegyptium* is able to transstadially pass in *A. phagocytophilum*. However, the probable lack of transovarial transmission of *A. phagocytophilum* in ticks [46] confers little eco-epidemiological importance to this vector-pathogen association.

The genus *Ehrlichia* includes five species [47], but only *E. canis* is found in Europe. This study reports the presence of *E. canis* for the first time in *H. aegyptium*, with a noteworthy prevalence. The only recognized vector for *E. canis* is *Rhipicephalus sanguineus*. In Romania, the distribution range of *H. aegyptium* overlaps with the distribution of *R. sanguineus*[19]. Although *R. sanguineus* feeds mainly on dogs [48], in Romania, it has been found also on hedgehogs (*E. roumanicus*) [19]. This is indicative of a possible cross transmission of *E. canis* from *R. sanguineus* to *H. aegyptium* using hedgehogs as bridging hosts.

The Q fever agent, *C. burnetii* was present only in Măcin Mountains with a relatively high prevalence in ticks (37.9%). An interesting aspect in this area was that all tortoises harbouring *C. burnetii*-infected ticks had also *A. phagocytophilum*-infected ticks and 72.7% of these tortoises had ticks infected with *E. canis*, too. Additionally, out of the 11 tortoises infested with *C. burnetii*-infected ticks, 10 (90.9%) had co-infected ticks. Q fever affects a wide range of domestic and free living mammals, birds, reptiles, fish, and arthropods, as well as humans [49,50]. The etiological agent of Q fever, *C. burnetii*, has been identified in over 40 tick species [50,51]. *Hyalomma aegyptium* was shown to have an unquestionable potential in the epidemiology of Q fever natural foci [18]. Ticks transmit *C. burnetii* vertically (transstadially and transovarially) and horizontally (by biting, via saliva) [52] but also through their faeces [53]. Sharing of pastures by tortoises and domestic ruminants was suggested by Široký et al. [18] to have an important role in the natural cycle, especially if considering that tortoises maintain natural foci of Q fever by hosting long-term infected ticks [18].

We found a marked dissimilarity between the occurrences of individual pathogens in ticks among the different locations: *Anaplasma phagocytophilum* and *E. canis* being found in all three locations while *C. burnetii* was only present in one. This variance may be caused by the different habitat associations and its host-fauna composition. The two forested sample sites have high occurrence rates of small mammals, especially hedgehogs, while the third location is primarily used by small domestic ruminants [54]. *Coxiella burnetii* is commonly reported in sheep and goats [55], hence its occurrence is more likely in the later habitat. This is consistent with the present findings. Moreover, the local agricultural practice (i.e. high turnover rate of domestic herds on extensive used pastures) in this region provides chances for a continuous presence of this pathogen in the environment [56].

## Conclusions

The presence and relatively high prevalence of three important zoonotic pathogens in *H. aegyptium* raises the question of their epidemiologic importance in disease ecology. As tortoises are unlikely reservoir hosts for *A. phagocytophilum* and *E. canis* and both these pathogens are common in *H. aegyptium*, this is an important indication for (1) a possible increased host-switching behaviour of these ticks to competent reservoir hosts (i.e. hedgehogs) and (2) transstadial transmission. Furthermore, if considering also the presence of *C. burnetii*, it can be concluded that *T. graeca* and its ticks should be evaluated more seriously when assessing the eco-epidemiology of zoonotic diseases.

## Competing interests

All authors have seen and approved the manuscript and declare that they have no competing interest.

## Authors’ contributions

PAI wrote the manuscript and made the statistical analysis; MIA and DMO identified the ticks; PAI, MIA and KZ performed the DNA extraction and PCR; MAD study design, MS concept and wrote the discussion, MAD, DG and SAD collected the samples; LM performed DNA extraction; GCM research project coordinator; CV team coordinator. All authors read and approved the final manuscript.

## Supplementary Material

Additional file 1 **Origin of samples of *Hyalomma aegyptium * used in this study.** Click here for file
