# Supplementary material for: Zoonotic pathogens associated with Hyalomma aegyptium in endangered tortoises: evidence for host-switching behaviour in ticks?
Source: Parasit Vectors. 2012 Dec 28;5:301. doi: 10.1186/1756-3305-5-301 (PMC3564739; doi:10.1186/1756-3305-5-301)
Supplement: Additional file 1 — Origin of samples of Hyalomma aegyptium used in this study. [file 1756-3305-5-301-S1.docx]

**Origin of samples of *Hyalomma aegyptium* used in this study**

| Region | Locality | Total number of ticks | Larvae | Nymphs | Males | Females |
| --- | --- | --- | --- | --- | --- | --- |
| **Măcin Mountains** | Pricopan | 125 | 1 | 12 | 88 | 24 |
|  | Măcin | 191 | 1 | 2 | 127 | 61 |
|  | Sulucu | 2 | 0 | 0 | 2 | 0 |
|  | Turcoaia | 15 | 0 | 0 | 10 | 5 |
| **Babadag forest** | Slava Cercheză | 1 | 0 | 0 | 1 | 0 |
|  | Codru | 28 | 0 | 0 | 19 | 9 |
|  | Movila golaşă | 19 | 0 | 0 | 14 | 5 |
|  | Babadag | 17 | 0 | 0 | 11 | 6 |
| **Constanţa forests** | Rasova | 24 | 0 | 0 | 17 | 7 |
|  | Canaraua Fetei | 13 | 0 | 1 | 7 | 5 |
|  | Dumbrăveni | 4 | 0 | 0 | 3 | 1 |
|  | Hagieni | 9 | 0 | 1 | 5 | 3 |
| **Total** | | **448** | **2** | **16** | 304 | 126 |
